# Are we degenerate tetraploids? More genomes, new facts

**DOI:** 10.1186/1745-6150-3-50

**Published:** 2008-12-10

**Authors:** Amir Ali Abbasi

**Affiliations:** 1 National Center for Bioinformatics, Faculty of Biological Sciences, Quaid-i-Azam University, Islamabad 45320, Pakistan; 2 Institute of Human Genetics, Philipps-University, Bahnhofstrasse 7, D35037 Marburg, Germany

## Abstract

**Background:**

Within the bilaterians, the appearance and evolution of vertebrates is accompanied by enormous changes in anatomical, morphological and developmental features. This evolution of increased complexity has been associated with two genome duplications (2R hypothesis) at the origin of vertebrates. However, in spite of extensive debate the validity of the 2R hypothesis remains controversial. The paucity of sequence data in early years of genomic era was an intrinsic obstacle in tracking the genome evolutionary history of chordates.

**Hypothesis:**

In this article I review the 2R hypothesis by taking into account the recent availability of genomic sequence data for an expanding range of animals. I argue here that genetic architecture of lower metazoans and representatives of major vertebrate and invertebrate lineages provides no support for the hypothesis relating the origin of vertebrates with widespread gene or genome duplications.

**Conclusion:**

It appears that much of the genomic complexity of modern vertebrates is very ancient likely predating the origin of chordates or even the Bilaterian-Nonbilaterian divergence. The origin and evolution of vertebrates is partly accompanied by an increase in gene number. However, neither can we take this subtle increase in gene number as an only causative factor for evolution of phenotypic complexity in modern vertebrates nor we can take it as a reflection of polyplodization events early in their history.

**Reviewers:**

This article was reviewed by Eugene Koonin, Joshua Cherry (nominated by David Lipman), and Jerzy Jurka.

## Background

To explain the genetic basis of major transitions in organismal evolution, in 1970 Susumu Ohno famously proposed that multiple rounds of whole genome duplications (2R hypothesis) had occurred during the early history of vertebrate lineage, driving the evolution of developmental and morphological complexity in vertebrates [1,2]. Ohno,s idea was based solely on genome size differences, chromosomal topologies, and recent tetraploidization events in some fish and amphibians. Over the past decade the 2R hypothesis has gained extensive popularity among evolutionary and developmental biologist.

Proponents presented several lines of evidence in favor of entire genome duplication hypothesis in the early vertebrates. First, compared to model invertebrate genomes (fruit fly, nematode, sea squirt and amphioxus) the typical vertebrate genome possess more genes [3]. Second, the existence of paralogons in the human genome [4,5]. Third, the conservation of gene synteny throughout vertebrates and their invertebrate ancestors and the spread of these anciently conserved syntenic fragments among multiple vertebrate chromosomes (vertebrate paralogons) [6,7]. Fourth, the refinement of extensive gene duplication events early in vertebrate history through molecular-clock based approaches (absolute dating) [8,9]. Fifth, the extrapolation of genome evolution scenarios in plant and yeast to genome evolution events in vertebrates [10-12]. A further piece of evidence in favor of two rounds of whole genome duplication hypothesis emerges from the observation that protostome invertebrates (fruit fly) and deuterostome cephalochordate amphioxus possess single HOX cluster whereas the vertebrates have four or more clusters [13-15]. Impressed by the fascination associated with this hypothesis some researchers have gone so far as to state " there is now incontrovertible evidence supporting the 2R hypothesis"[16].

Opponents of the 2R hypothesis argued that the current data is not compelling evidence of polyploidization and increase in the number of paralogous genes in vertebrates occurred as a result of small scale gene duplication events involving single genes and chromosomal segments, scattered at different times during the history of life [17-21].

**Figure 1:**
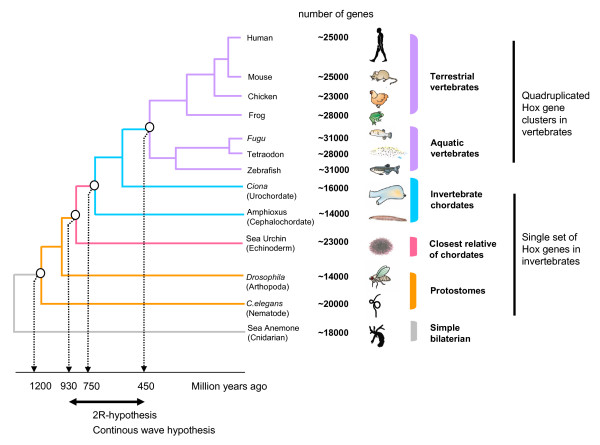
**A phylogeny of animals**. Evolutionary relationship, numbers of genes and divergence times of selected animals whose genomes have been sequenced. Along with the increasing overall gene number, the occurrence of single HOX gene cluster in invertebrate genome and four or more clusters in vertebrate genome is taken as evidence for two rounds of whole genome duplication at the base of vertebrate lineage (2R hypothesis). The double headed arrow underneath shows the proposed time windows of genome amplification events. Features not drawn to scale.

In spite of extensive empirical scrutiny, the 2R remains controversial. The intrinsic difficulty was the lack of broad phylogenetic representation in the available sequence data. However, the recent availability of sequence data from an expanding range of vertebrate and invertebrate species from interspersed time points (Figure 1) has provided an unprecedented opportunity to test two main competing hypotheses by employing the deepest and more diverse sampling of organisms.

## Presentation of the hypothesis

### Rapid paralogous gene increase in the early stage of vertebrate evolution

The recently sequenced genomes for two morphologically simple invertebrates, sea anemone (a cnidarian model) and sea urchin (nonchordate deuterostome) provides a unique perspective on the evolution of animal genomes [22,23]. The genomic complexity of sea anemone and sea urchin, in terms of gene contents and structure, suggests that many features of the genetic toolkit seen in modern vertebrates existed in the ancestral deuterostome genome or even in the common ancestor of bilaterians-nonbilaterians animals (Figure 1). Surprisingly this ancestral animal gene set has undergone extensive contraction in many protostomes and nonvertebrate chordate animals. Many gene families that were expanded deep in the history of life (before the bilaterian-nonbilaterian divergence) and whose descendants survive in modern vertebrates, appeared to be absent in model protostomes (fruit fly, nematode) and chordates (amphioxus, *Ciona*). For instance, *Nematostella vectensis *(sea anemone) which is a model animal from the basal metazoan phylum Cnidaria shares 11 of 12 *Wnt *genes found in humans, while fruit fly shares only 6 of 12 [24]. Similarly, the number of orthologous genes between the ascidian (nonvertebrate chordate) and the modern vertebrates are less than the number estimated between the vertebrates and the sea urchin (simple deuterostome) [23]. Inferring gene/genome duplication events at the base of vertebrate lineage, by using any of these highly derived invertebrate model outgroups, including amphioxus which was previously assumed as a good representative of ancestral chordate genome, would thus be problematic. For instance, defining the duplication time windows for human gene families (under the 1R/2R hypothesis) by using an invertebrate outgroup such as amphioxus/ciona or fruit fly could falsely place many of those gene expansion events that might have occurred deeper in the life history, at the base of vertebrate lineage [9,25]. This sort of data could inaccurately show a rapid paralogous gene increase in the early stage of vertebrate evolution and could also lead to a misleading conclusion of big-bang events (genome duplications).

### Morphological complexity and gene number

The homeobox genes are key components of developmental toolkit of animals, and it has been suggested that functional diversification of these genes through duplication and divergence was pivotal in the evolution of complex developmental and morphological features in bilaterian animals. If the origin of particular homeobox gene is in actuality associated with the evolution of particular animal trait, in that case one should expect an increase in the homeobox toolkit repertoire during evolutionary transitions from simple to complex life forms, like from basal diploblastic animals to triploblastic life forms, protostome to deuterostome and from simple chordates to modern vertebrates. However, recent data shows that all the major classes of homeodomain coding genes have undergone significant radiation deep in animal evolution, before the divergence of Cnidaria and Bilateria [26]. Surprisingly, the genome of morphologically simple nonbilaterian, the sea anemone possesses significantly more homeobox genes than the morphologically and developmentally complex fruit fly.

Furthermore, a fundamental problem with the hypothesis linking the sudden appearance of complex morphological traits in vertebrates with whole genome/extensive gene duplication events is that in defining the evolutionary steps in the phenotypic complexity, the extinct lineages were completely ignored. However, when fossils are taken into account, jumps in morphological complexity disappear and the proposed whole genome duplication in vertebrate history no longer is correlated with the origin of body plans [27]. Thus it appears that a correlation between the big jumps in the morphological complexity and proposed whole genome duplication in vertebrates is an artifact of incomplete taxonomic sampling.

Given the fact that there is no apparent relationship between the gene number and organismal complexity (Figure 1), the assumption that modern vertebrates have attained higher level of complexity in their body plans by expanding their genetic toolkit through big-bangs of genome amplification events is not credible. Instead the most plausible explanation of organismal complexity is to deploy each gene with particular developmental function at many times and places during development. This increase in the diversity of gene function may involve an increase in the number of transcription factors or an increase in the number of *cis*-acting regulatory elements that control region-specific expression of genes[28]. This sort of regulatory evolution creates many-fold increase in the complexity of developmental processes without drastic expansion of gene number [29-32].

### Paralogy regions in the human genome

The distinct chromosomal regions within a genome that contains a set of similar genes are known as paralogy regions or paralogons. Very often the genetic constitution of these intra-genomic homology blocks remains conserved across distantly related genomes (conserved synteny) [6,7,33]. The occurrence of two-fold, three-fold and four-fold paralogy regions in human and other vertebrates and their co-occurrence in distinct genomes is taken as an evidence that ancestral genome of modern vertebrates was shaped by two rounds of polyploidization (2R hypothesis) [4,6,25,34-36]. Alternative hypothesis suggests that the constituent multigene families of these anciently conserved vertebrate paralogy regions arose as a result of small scale duplications involving gene clusters and chromosomal segments which occurred at different time points during chordate evolution [17,37-39]. Whereas the spread of similar gene sets on distinct vertebrate chromosomes is the consequence of subsequent rearrangement events[17,40]. The conservation of these blocks of genes both within and between species may reflect selective reasons [37]. For instance; to have genes of similar expression or function in the physical proximity on a chromosomal segment.

In fact, the occurrence of two-fold paralogy regions can be explained by many alternative explanatory scenarios, whereas the three and four-fold paralogy regions may be indicative of two rounds of ancient whole genome or whole chromosome duplication events. However, given the large size of human and other vertebrate chromosomes the fraction of gene families having three or four representatives on four-fold paralogy regions is very small [41]. For instance, human HOX cluster bearing chromosomes (Hsa2, 7, 12 and 17) contains in total ~4609 known coding genes (NCBI 36 assembly of human genome), whereas the HOX cluster paralogon harbors only 11 known multigene families with at least three of their members anciently linked to HOX clusters [17]. This illustrates that spatial organization of ~0.95% of coding contents of human HOX cluster bearing chromosomes favors the structuring of these chromosome through ancient tetraploidization events, whereas the history of remaining portion may not be in harmony with this scenario. Thus it is not plausible to take the presence of few similar gene sets on three or four human chromosomes as evidence that the chromosomes are related by large duplication.

Moreover, for identifying ancient paralogons in the vertebrate genomes some researchers have combined the map-self comparison approach with a comparison with a pre-duplication species. In these studies the coverage and distribution pattern of old paralogs [5] or anciently conserved syntenic fragments [6] in the human and other vertebrate genomes was subjected to statistical analysis to rule out the null hypothesis: that the constituent gene families of vertebrate paralogons have arisen by distinct duplication events and their members were brought together in three or four collinear regions on different chromosomes as a result of rearrangement of genomic segments including multiple contiguous genes [17,37]. However, it is advisable to recognize that the statistical validation of distribution of old paralogs or anciently conserved (vertebrate-invertebrate) syntenic segments among multiple vertebrate chromosomes does not constitute the evidence for the mechanism of origin of paralogons. Therefore, special care should be taken in interpreting the sheer map distribution of a subset of ancient vertebrate genes as an illustration of polyploidization in vertebrate early evolutionary history.

The precise nature of those events that has created ancient paralogy regions (> 450 Mya) of vertebrate genome is hard to track because such events are obscured by long term evolutionary divergence and rearrangements. However, an important insight into these ancient events can be gained by tracing the genome evolutionary scenario of very recently diverged species. For instance, the availability of genome sequence data for multiple primate species has shown that that our own genetic material is expanded by ~5% during the past 35–40 million years (divergence of New and Old World monkeys) of evolution through segmental duplications (SDs) and rearrangement events [42,43]. These recent human segmental duplications are large nearly identical copies of genomic DNA, ranging from 300 kb to 1 Mb in size, and are present in at least two genomic locations [44]. Detailed analysis has attributed several roles to SD events: creation of new genes in primates, expansion of multigene families, and triggering large scale chromosomal rearrangements in hominoid [44]. Huge impact of recent SDs in architecturing the primate genome over the short period of evolutionary time, apparently support the notion that small scale duplications and rearrangements might have remained a predominant mechanism in shaping the vertebrate genome throughout their evolutionary history [37,38].

Now the question is that, what kept the similar set of genes together on distinct chromosomal regions or in distinct genomes? The preservation of intra-genomic and inter-genomic synteny over longer period of evolutionary time is unlikely to be the result of chance. Empirical data strongly support the notion that, there are adaptive reasons to keep certain configurations of genes together over evolutionary time [37]. For instance, higher order structural organization of chromosomes, might help in co-regulation of particular sets of neighboring genes in a coordinated manner by recruiting them to shared regions of gene expression [45]. Similarly, the gene regulatory elements spread across long regions impose critical constraint on genomic architecture and are known to have maintained exceptionally long syntenic blocks both within and across species [46-48].

### HOX cluster duplication and the history of vertebrate genome evolution

**Figure 2:**
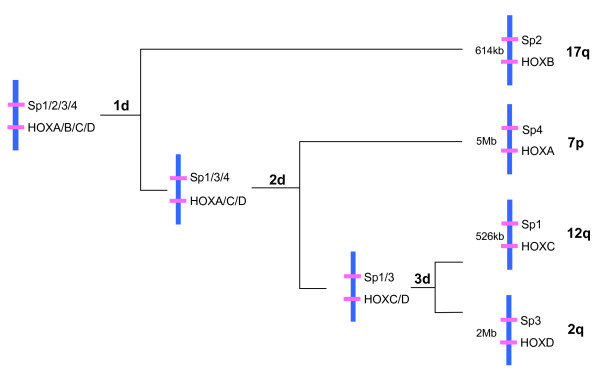
**Evolutionary history of Human HOX clusters and closely linked SP family members**. Close physical linkage and congruency in the phylogenies of human SP gene family members and HOX clusters supports the view that vertebrate HOX gene clusters arose as a result of small scale gene duplication events involving chromosomal segments or gene clusters. 1d-3d, three rounds of segmental duplication. Features not drawn to scale.

HOX family of homeobox containing transcriptional factors lies in adjacent positions and forms a cluster along a chromosome in animal genomes. All invertebrates including fruit fly and amphioxus have only one HOX gene cluster whereas all vertebrates possess more than one set of HOX clusters [49]. The fact that human and other mammals possess four coherent HOX gene clusters whereas their closest invertebrate relative amphioxus contain just a single set of collinear HOX genes, is taken as evidence that the origin of vertebrates coincided with two rounds of whole genome duplication events [10,50]. Undoubtedly the HOX gene cluster duplication occurred specifically on the vertebrate lineage [13,51]. However, conclusions regarding HOX gene clusters alone cannot to be extended to define the evolutionary history of entire genome. In fact the evolutionary history of human HOX clusters itself support the notion that they originated by regional duplication. Zhang and Nei [52] analyzed the phylogeny of mammalian HOX clusters and proposed two alternative topologies (((HOXC HOXD) HOXA) HOXB) and ((HOXC HOXD) (HOXA HOXB)). The former topology suggests three separate regional duplication steps (1→2→4 HOX clusters) whereas the later favors two rounds of whole genome duplication events (2 + 2 topology). Analysis of COL and ERBB multigene families, whose members are closely linked to four human HOX clusters, did not resolve the cluster duplication events decisively [37,53]. The representatives of human SP gene family are very closely spaced to HOX clusters, with human SP1 gene mapping at approximately 526 kb centromeric to HOXC, SP2; at ~614 kb centromeric to HOXB, SP3; at ~2 Mb centromeric to HOXD and SP4; at ~5 Mb telomeric to HOXA (Figure 2). The close physical linkage of SP family members with each of human HOX clusters makes them an interesting test case to evaluate the HOX cluster duplication history. Thorough phylogenetic analysis of SP family members (employing broad taxonomic sampling) has revealed that SP1, SP2, SP3 and SP4 genes share their duplication history with HOX clusters and have arisen together with the HOX clusters through three separate segmental duplication steps [17] (Figure 2). Thus congruency in the phylogenies of human HOX clusters and SP genes not only resolved the cluster duplication events but also decisively rejected the assumption that four HOX clusters in tetrapods are the result of two rounds of whole genome duplication early in vertebrate history (Figure 2).

## Testing the hypothesis

I have questioned the validity of arguments adduced in support of Ohno's view that the complexity of vertebrate genomes originated by means of whole genome duplications. Instead, the hypothesis presented here suggests that the animal genomes evolved through small-scale duplication events scattered at different times over the history of life. This hypothesis is testable in the light of currently available extensive genomic data form an expanding range of vertebrate and invertebrate animals: 1) through comparison of total gene number and gene family size differences among modern vertebrate and basal invertebrate (for instance, sea anemone/sea urchin) genomes; 2) by estimating the fraction of vertebrate chromosomes occupied by three or four fold paralogy regions; 3) by conducting the phylogenetic analysis of multigene families with three or more of their representatives residing on four-fold paralogy regions (paralogons) in the human genome; 4) by estimating the genome evolutionary scenario of very recently diverged vertebrate species (for instance, primates among vertebrates). Evaluating the correlation between the organismal complexity and gene duplications may also be helpful in testing the validity of two main competing hypotheses [27] (small-scale duplication versus 2R hypothesis).

Very often the statistical evaluation of vertebrate genome evolutionary history is based on comparative data from few vertebrate and highly derived invertebrate genomes and thus could inadvertently lead to unfounded conclusions. Therefore, I propose that broadening of taxonomic sampling in statistical approaches by employing the currently available extensive genomic data, in particular from well preserved basal metazoan genomes and recently diverged vertebrate species, would provide a much clearer picture of animal genome evolution.

## Implications of the hypothesis

Initial attempts to unravel the evolutionary aspects of our own genome have borne out highly controversial results. Some has suggested that drastic events (2 rounds of whole genome duplication/2R hypothesis) at the base of vertebrate lineage led to the greater genetic complexity in modern vertebrate genome whereas other researchers rejected the 2R hypothesis at all and suggested a continuous mode of small scale duplications (segmental/gene cluster). These alternative scenarios of vertebrate genome evolution are largely based on data from human and few other vertebrate and invertebrate genomes. The recent availability of additional vertebrate and invertebrate genomes has provided an unprecedented insight into the core evolutionary processes that had shaped our genome, deep in the history of life. Surveying of newly sequenced genomes from the deepest branches of life has revealed that many components of the genetic toolkit seen in modern vertebrates arose and diversified deep in animal history even before the origin of chordates. Indeed the origin and evolution of vertebrates is partly accompanied by an increase in gene number, for instance one coherent HOX cluster in amphioxus like invertebrate to four or more HOX clusters in modern vertebrates. However, neither can we take this subtle increase in gene number as an only causative factor for evolution of phenotypic complexity in modern vertebrates nor we can take it as a reflection of whole genome duplication events early in their history. In depth analysis of the genomic data from recently diverged primate species on markedly different phenotypic trajectories provides valuable clues to ancient genomic events. These data supports the notion that small scale duplications and rearrangements have remained a pervasive phenomenon, driving the vertebrate evolution both at phenotypic and genotypic level, throughout their history. I conclude, therefore, that the comparative genomic data from species that diverged early in metazoan evolution (such as Cnidarian-bilaterian) as well as from the very recently diverged animals (such as primates among vertebrates) provides no evidence in favor of the ancestral tetraploidy in vertebrates. In fact it appears that the 2R hypothesis is an artifact, invoked by the lack of phylogenetic breadth in the genome sequence data in early years of genomic era.

## Competing interests

The author declares that they have no competing interests.

## Authors' contributions

AAA conceived and designed the project. AAA wrote and approved the final manuscript.

## Reviewers' Comments

### Reviewer report 1

Dr. Eugene V. Koonin

National Center for Biotechnology Information

National Library of Medicine

National Institute of Health

**Bethesda, USA**.

This is a critical overview of the 2R hypothesis (two rounds of whole genome duplication) on the origin of vertebrates. The conclusion that, to a large extent, is based on the unexpected genomic complexity of organizationally simple animals, such as sea anemone, and on the modest number of 2-fold and, particularly, 4-fold paralogons in vertebrate genomes, is that there is currently no basis to accept the 2R hypothesis. Instead, it is proposed that the vertebrate genome evolved by relatively small, regional duplications.

This is an old controversy to which this paper does not add any new analysis, only discussion, and that, in my opinion, somewhat perfunctory. As I see the situation, the jury is still out with regard to the 2R. It is important to realize that the 2R hypothesis has gone a long way since the days it was first proposed by Ohno. The 2R hypothesis now claims support from the comparisons of the gene order in the vertebrate paralogons and in Amphioxus, i.e., the duplications in vertebrates appear to be synteny-preserving rather than synteny-disrupting [6]. Even more importantly, perhaps, a global analysis of the distribution of old paralogs in vertebrate genomes has been claimed to support 2R [5,16]. Thus, 2R is not sheer speculation, considerable effort has been undertaken to test this hypothesis, and there are strong claims of evidence that consistently supports it.

Personally, I have a certain epistemological sympathy for the position taken in this paper in the sense that I believe that, as a matter of principle, the piecemeal duplication model should be the null hypothesis of (in this case, vertebrate) genome evolution that has to be falsified in favor of WGD scenarios. I doubt that the current statistical argument for such falsification is overwhelming so that 2R is to be accepted as the final verdict. However, I also think that the evidence in support of 2R is rather diverse and rather substantial, so it needs to be addressed seriously rather than summarily dismissed, primarily, on the basis of the high complexity of primitive metazoan genomes which is not a logically consistent argument against 2R. I believe that, for a really critical assessment of the 2R hypothesis, the evolutionary genomics literature, and in particular, the evidence claimed in support of 2R should be examined in considerably greater detail and more carefully, with special attention to the underlying assumptions of the statistical models employed in the respective studies.

#### Author Response

I am thankful to Dr. Koonin for his comments on this manuscript.

**1**. It is advisable to recognize that the statistical support for the spread of old paralogs or anciently conserved (vertebrate-invertebrate) syntenic fragments among multiple vertebrate chromosomes [5,6] does not constitute the evidence for the mechanism of origin of vertebrate paralogy regions. I propose therefore, that, special care should be taken in interpreting the sheer map distribution of a subset of ancient vertebrate genes as a strong support of polyploidization in vertebrate early evolutionary history.

**2**. In this article I do not intend to challenge the statistical models describing the vertebrate genome evolutionary events. My purpose here is to highlight the fact that well preserved genetic architecture of basal metazoans and comparative analysis of primate genomes (or any other group of vertebrates with comparable relatedness) casts serious doubt on the plausibility of the 2R hypothesis. Instead the recently sequenced genomes of animals from interspersed time points clearly shows that much of the genomic complexity seen in the modern vertebrates is very ancient than was previously anticipated (by 2R proponents). It appears that vertebrates had accomplished this genomic complexity through piecemeal duplications at widely different times over the evolution of life.

Statistical testing of vertebrate genome evolutionary scenarios is often based on comparative observations from few vertebrate and highly derived invertebrate genomes, and thus could inadvertently lead to unfounded conclusions. Therefore, I recommend that future statistical approaches to test hypothesis concerning vertebrate genome evolution, should take into account the newly sequenced genomes of basal metazoan animals and recently diverged vertebrate species (for instance primates).

**3**. In the light of your comments I have considerably expanded the survey of evolutionary genomics literature.

### Reviewer report 2

Dr. Jerzy Jurka

President & Director

Genetic Information Research Institute

**Mountain View, USA**.

The author presents a critical review of the so-called "Ohno's hypothesis" or "2R hypothesis" postulating that the early vertebrate lineage underwent one or more complete genome duplications. The author argues that the genome sequence data do not support the 2R hypothesis. While I am not sure if the 2R hypothesis is falsifiable based on genomic data, I would support this publication if the author could include discussion of a recent paper in favor of the 2R hypothesis.

Masanori Kasahara, "The 2R hypothesis: an update", "Current Opinion in Immunology" (2007), doi:10.1016/j.coi.2007.07.009

#### Author response

I am thankful to Dr. Jurka for reviewing this manuscript.

In the revised manuscript, by keeping in view the suggestion of Dr. Jurka, I included the recent paper from Kasahara M. (2007) [16] and other articles favoring 2R.

### Reviewer report 3

Dr. Joshua L. Cherry

National Center for Biotechnology Information

National Library of Medicine

National Institutes of Health

**Bethesda, USA**.

**Nominated by David J Lipman, National Center for Biotechnology Information, NIH, Bethesda, USA**.

This review article assesses the hypothesis of two whole-genome duplications in vertebrate evolution in light of recent sequence data. I agree with the article's conclusion that there is no good reason to believe this hypothesis. I have a few comments about some of the arguments presented and the implications of some of the language used.

I found the role of the SP gene family in resolving the history of HOX clusters to be unclear. In fact the argument is more tenuous than the discussion would suggest. How can phylogenetic analysis of SP reveal both that the SP genes "share their evolutionary history with HOX clusters" and that HOX genes have "arisenthrough three separate segmental duplication steps"? Knowing that the evolutionary histories are the same would presumably entail knowing the phylogeny of HOX, so that the SP phylogeny would provide no additional information about HOX. It is in fact simply assumed that the candidate HOX phylogeny that agrees with the SP phylogeny is the correct one. This is possible, but if other linked paralogs have different histories, as suggested by the cited references, it is far from certain.

I would add that it is too strong to say that the alternative rooted topology, ((HOXC HOXD) (HOXA HOXB)), "favors two rounds of whole genome duplication events". This topology is consistent with 2R, but also with three local duplication events. In the absence of whole-genome duplications it would not be surprising to find some sets of paralogs with this type of topology.

I am uncertain of the meaning, in paragraph 3 of Paralogy Regions in the Human Genome, of "those vertebrate species that have recent evolutionary origin." Because the species analyzed are primates, this might be taken to imply, incorrectly, that humans and our closest relatives are more recently evolved than other organisms and that evolution is a thing of the more distant past for other groups. Comparative analysis of primates can of course yield valuable information, but the same role could be played here by any other group of vertebrate species with comparable relatedness. Other expressions in the manuscript also suggest a ladder-like view of evolution, even if that is not the author's intent: "genetic architecture of deepest as well as most recent branches of animals" (Abstract); "evolutionary basal invertebrate" (paragraph 1 of Rapid Paralogous Gene Increase); "an ideal chordate ancestral genome" (same paragraph); "the deepest branches of life" (final paragraph).

#### Author response

I am grateful to Dr. Cherry for valuable comments and useful suggestions on this manuscript.

**1**. The most parsimonious explanation of the order of branching in HOX cluster and closely linked SP phylogenies is that, both of these gene families arose simultaneously (co-duplicated group) through three independent duplication steps (Figure 2). Other genes families (having three or four members linked to HOX clusters) in the HOX cluster paralogons, e.g. ERBB, COL, GLI, HH, SLC4A and others have recently been resolved into four discrete co-duplicated groups [17]. It has been shown that genes within each of these co-duplicated groups (of HOX cluster paralogons) are duplicated in concert with each other whereas the constituent genes of two different co-duplicated groups may not have duplicated simultaneously [17]. This observation is contrary to 2R scenario, which assumed that constituent gene families of HOX cluster paralogons arose simultaneously through two rounds of WGD.

**2**. I must agree that the SP phylogeny helps in understanding the phylogeny (duplication history) of HOX and would provide no additional information about HOX evolutionary history. Therefore, I replaced the term "share their evolutionary history" with the "share their duplication history" (HOX cluster duplication and the history of vertebrate genome evolution).

**3**. I must also agree with Dr. Cherry's argument that in the absence of whole-Genome duplications it would not be surprising to find some sets of paralogs with (AB)(CD) type topology.

**4**. In the revised manuscript I tried to erase a ladder-like view of evolution and have used precise terms to explain the evolutionary relatedness among animals.

**5**. Minor issues were also addressed in the revised manuscript.
